# Assessing sex-differences and the effect of timing of vaccination on immunogenicity, reactogenicity and efficacy of vaccines in young children: study protocol for an individual participant data meta-analysis of randomised controlled trials

**DOI:** 10.1136/bmjopen-2016-011680

**Published:** 2016-07-29

**Authors:** Merryn Voysey, Andrew J Pollard, Rafael Perera, Thomas R Fanshawe

**Affiliations:** 1 Nuffield Department of Primary Care Health Sciences, University of Oxford, UK; 2 Oxford Vaccine Group, Department of Paediatrics, University of Oxford, and the NIHR Oxford Biomedical Research Centre, UK

**Keywords:** Sex-differences, vaccine, immunogenicity, individual participant data, meta-analysis

## Abstract

**Introduction:**

Disease incidence differs between males and females for some infectious or inflammatory diseases. Sex-differences in immune responses to some vaccines have also been observed, mostly to viral vaccines in adults. Little evidence is available on whether sex-differences occur in response to immunisation in infancy even though this is the age group in which most vaccines are administered. Factors other than sex, such as timing or coadministration of other vaccines, can also influence the immune response to vaccination.

**Methods and analysis:**

Individual participant data meta-analysis of randomised controlled trials of vaccines in healthy infants and young children will be conducted. Fully anonymised data from ∼170 randomised controlled trials of vaccines for diphtheria, tetanus, *Bordetella pertussis*, polio, *Haemophilus influenzae* type B, hepatitis B, *Streptococcus pneumoniae*, *Neisseria meningitidis*, measles, mumps, rubella, varicella and rotavirus will be combined for analysis. Outcomes include measures of immunogenicity (immunoglobulins), reactogenicity, safety and disease-specific clinical efficacy. Data from trials of vaccines containing similar components will be combined in hierarchical models and the effect of sex and timing of vaccinations estimated for each outcome separately.

**Ethics and dissemination:**

Systematic reviews of published estimates of sex-differences cannot adequately answer questions in this field since such comparisons are never the main purpose of a clinical trial, thus a large degree of reporting bias exists in the published literature. Recent improvements in the widespread availability of individual participant data from randomised controlled trials makes it feasible to conduct extensive individual participant data meta-analyses which were previously impossible, thereby reducing the effect of publication or reporting bias on the understanding of the infant immune response.

Preliminary results will be available in 2016 with final results available in 2019. No ethics review is required for secondary analyses of anonymised data.

**Strengths and limitations of this study**

- A very large number of studies is available for inclusion in the meta-analysis.
- A central laboratory was used for most studies resulting in standardisation and consistency of measurements and removing interlaboratory variation.
- Studies conducted in varied locations throughout the world which enhances the generalisability of findings.
- Robust analysis methods using mixed-effects models.
- All studies are from one company therefore there are few studies which compare vaccines from different manufacturers.

## Background

### Sex-differences

Males and females have different levels of risk for certain diseases. For example, women are more likely to develop multiple sclerosis than men^1^ and in young children, pneumonia and meningitis more often occur in boys than girls.^2–4^ In the same way that differences between the sexes are observed for some infectious diseases, differences may also occur in their immune responses to vaccination. The biological mechanisms by which males and females respond differently to vaccines are multifactorial and not well understood.^5^ ^6^ Females have two X chromosomes which contain many genes related to immune mechanisms. Males and females also have different hormone levels which, additionally, change over time, further affecting a person's ability to respond to a vaccine or other source of immune challenge. Differences between males and females in response to vaccination have been mostly observed for viral vaccines in adults; however, the number of studies in which sex-differences in vaccine responses are reported is very small in comparison to the number of vaccine trials conducted.^6^ Even less information is available as to whether young boys and girls respond differently to their vaccinations, even though the majority of vaccines are administered to those in this early age group.

We have previously shown in a meta-analysis of a small number of studies^7^ that responses to some serotypes of pneumococcal and meningococcal vaccines are higher in girls than boys, but that there are no differences in responses between girls and boys for *Haemophilus influenzae* type B or tetanus vaccines. In this project we expand on that work and assess sex-differences in response to many vaccines administered to infants and young children. Data from ∼170 randomised controlled trials of vaccines in young children will be accessed which will enable a comprehensive assessment of differences in response to vaccines between girls and boys. If clinically relevant sex-differences in responses to vaccines exist, then it may be possible to tailor vaccine doses to specific sexes. In addition, if substantial differences between the sexes exist for some vaccines, licensing of future new vaccines for those antigens may require sufficient immunogenicity to be demonstrated in the sex known to have poorer responses.

### Vaccine schedules

The timing of infant vaccination schedules can vary from country to country. In the UK children are currently vaccinated at 2, 3 and 4 months. Many other countries vaccinate at 2, 4 and 6 months; Sweden, Austria, Norway and Italy recommend a two dose infant schedule at 3 and 5 months. The WHO recommends a 6-week, 10-week and 14-week schedule for diphtheria–tetanus–pertussis (DTP) vaccines.^8^ Schedules with a later first dose, wider spacing of doses and, counterintuitively, schedules with fewer doses in early infancy followed by a booster dose for toddlers, may result in similar immunogenicity in the first 6 months of life and better immunogenicity after the booster dose.^9^ Few trials compare immune responses in children who have been vaccinated under different schedules. It is the timing of the initial priming doses given in infancy which can vary between countries and the timing of delivery of a booster vaccination to toddlers and both may impact on the child's ability to mount a good immune response.

### Hypotheses for investigation

#### Sex-differences

1. For which vaccines, and at which ages/time points does immunogenicity differ between girls and boys?

Subgroup hypotheses

- Does coadministration of live attenuated viral vaccines impact on differences between girls and boys in their response to the non-viral vaccines?
- Does prior administration of other vaccines (eg, BCG) impact on differences between girls and boys in responses to viral or non-viral vaccines?
- Do sex-differences in immune responses to bacteria protein conjugate vaccines differ according to the type of conjugate protein?
- Do sex-differences in immune responses to bacterial conjugate vaccines with diphtheria carrier proteins cause similar differences in responses to coadministered diphtheria toxoid vaccine?

- For which vaccines, and at which ages/time points does reactogenicity differ between girls and boys?
- Are observed sex-differences in immune responses associated with sex-differences in clinical efficacy?

Timing of vaccination

- What difference in immunogenicity or reactogenicity can be attributed to differences in the spacing of doses in the immunisation schedule in infants? (eg, 2, 3 and 4 months schedules compared with 2, 4 and 6 months schedules)
- Does the age at which a priming or booster vaccine is given or the length of time between the priming and booster vaccines, affect vaccine-antigen-specific immunogenicity or reactogenicity?

## Methods

### Study design

#### Types of studies

Randomised controlled trials assessing the immunogenicity, safety and efficacy of vaccines in infants and healthy young children.

Studies will be excluded which:

- Enrolled preterm infants or children with comorbidities;
- Enrolled a majority of children over the age of 3 years;
- Did not measure immunological responses (trials of efficacy or safety only).

#### Types of interventions

The following vaccines, administered as part of a trial as either the randomised intervention or as a coadministered routine vaccine will be included. Monovalent vaccines administered separately and combination vaccines will both be included.

1. Diphtheria toxoid
2. Tetanus toxoid
3. Pertussis (acellular or whole cell)
4. Polio (inactivated or oral)
5. Hepatitis B
6. *H. influenzae* type B
7. Streptococcus pneumoniae
8. Neisseria meningitidis
9. Rotavirus
10. Measles
11. Mumps
12. Rubella
13. Varicella.

### Types of outcomes for analysis

#### Primary outcome

1. Immunogenicity: vaccine antigen-specific antibody responses measured at 1-month postpriming, prebooster and 1-month postbooster and persistence at further postbooster time points if available.

#### Secondary outcomes

1. Reactogenicity: solicited local and systemic reactions measured by participant diary within 7–10 days postvaccination:

- Local reactions (erythema, induration, swelling);
- Systemic reactions (pain, fever, irritability, loss of appetite).
- Efficacy: vaccine-related disease incidence as defined in study protocols.
- Safety: serious adverse events.

### Additional data to be collected

Individual-level data:

1. Sex
2. Age at enrolment (to nearest week)
3. Age at each visit (to nearest week).

Study-level data:

1. Laboratory
2. Assay type (commercial or inhouse)
3. Date the study started
4. Investigational and concurrent routinely administered vaccines.

Country-level data:

1. Country
2. BCG vaccination given at birth.

### Data source

Anonymised individual participant data and associated study documents from ∼170 randomised controlled trials will be accessed through https://clinicalstudydatarequest.com/. We reviewed all trials in which the medicine listed for the study was one of the vaccines meeting our inclusion criteria. The study listing was accessed to determine if the characteristics of the trial met the inclusion criteria for the study and if so, the trial was added to the data request. All studies were sponsored by GlaxoSmithKline, thus providing a homogenous data source in which study procedures, study documentation, data management and laboratory processes are similar across studies. No other pharmaceutical company registered on https://clinicalstudydatarequest.com/ has substantial numbers of vaccine studies available for additional research, and so these were excluded to ensure methodological consistency.

### Data synthesis methods

Meta-analysis is a well-established method of combining information from multiple clinical trials or observational studies to obtain more precise estimates of treatment effects or differences.

#### Immunological endpoints

Immunological measures such as antibody concentrations are usually log-normally distributed and thus will be log-transformed prior to analysis. Data from multiple studies of vaccines containing the same antigens will be combined and all participants with at least one measure of postvaccine immunogenicity will be included in the analyses. In order to ensure that all immunological responses compared are responses to the vaccine antigen of interest, participants who did not receive the vaccine antigen of analysis or who received a placebo will be excluded from immunogenicity and reactogenicity analyses.

For the main analysis of sex-differences in immunological responses (hypothesis 1), a two level hierarchical mixed-effects model will be used which includes a study-specific random intercept and fixed effects for sex and type of vaccine received. Additional fixed effects for schedule (timing) of administration, country and age at enrolment will be explored and included in the model if inclusion improves model fit (using Akaike's information criteria). Where multiple time points are measured on the same participant and can be combined in one model, a 3-level hierarchical model will be used with the appropriate additional random intercept for each participant. The antilog of the parameter estimate for the effect of interest will be presented as a geometric mean ratio with associated 95% CI.

Subgroup comparisons will be used to assess factors which influence sex-differences in responses (hypotheses 1—A,–D) by including within the model, an additional subgroup-by-sex interaction term.

#### Timing of vaccination (hypotheses 4 and 5)

The impact of the timing and spacing of the primary vaccination schedule in infancy on immunogenicity will be assessed by the inclusion of the spacing of doses and the age at first vaccination as fixed effects in the models described above. Additionally, the impact of increasing the length of time between priming and boosting doses of vaccines will be assessed along with the age at booster vaccination, by testing these variables in the model. The relationship between age at booster or spacing between prime and boost vaccines may not be linear and so the functional form of the relationship between this variable and immunogenicity outcomes will first be assessed graphically to determine if the linear assumption of the model is valid. Models will be adjusted for baseline (prevaccination) antibody levels.

#### Reactogenicity end points

Binary reactogenicity endpoints such as the presence or absence of fever, as well as other non-continuous endpoints will be analysed using a similar approach with generalised linear mixed-effects models (hypotheses 1 and 2).

#### Efficacy endpoints

Unlike trials of immunogenicity, trials in which the clinical efficacy of a vaccine is assessed involves long-term follow-up of participants to measure the incidence of clinical disease in the active vaccine and the placebo-control groups. Thus for the assessment of sex-differences in clinical efficacy (hypothesis 3), all participants (including placebo participants) will be included in these analyses. For each disease-specific efficacy outcome, sex differences in immune responses in the placebo and relevant vaccine arms will be computed separately and then the difference between the two estimates compared using a model which includes an additional term for the vaccine group-by-sex interaction effect. This will be interpreted in light of any sex-differences observed in the immunogenicity of the vaccine arm of the trial.

#### Statistical power

The substantial amount of data available for this study will result in very highly powered analyses. Very tight CIs around parameter estimates are expected, in particular for continuous immunological endpoints, resulting in conventionally statistically significant results which may not be of clinical relevance. We will report all results of all planned subgroups and outcomes with CIs rather than p values, however only results which are of statistical and clinical relevance will be considered important.

#### Heterogeneity

The extent of heterogeneity will be determined by estimating the between-study variance from hierarchical models.

### Limitations

All studies in these meta-analyses are from a single manufacturer and while this provides some benefits, such as similar laboratory processes, there are limitations to this approach. Findings based on meta-analyses of vaccines from a single manufacturer are not always applicable to vaccines from other manufacturers. In addition, data for some variables, such as location and BCG status, are only available at country level rather than individual level. This makes comparisons at country-level possibly open to some confounding.

### Ethics and dissemination

Preliminary results will be available in 2016 with final results available in 2019. No ethics review is required for secondary analyses of anonymised data.

## Discussion

Questions surrounding the differential response to vaccination in infants and children cannot be adequately addressed from reviews of the published literature. Quantifying sex-differences in immune response is never the primary aim of a vaccine clinical trial and it is not good practice in clinical trial reports to assess additional subgroup comparisons that are not prespecified in the protocol. Thus published reports of sex-differences usually occur only when statistically significant differences have been found, and for this reason substantial opportunity exists for introducing publication and reporting biases into systematic reviews. Individual participant data meta-analyses using original trial data sets are a more reliable approach than a systematic review of previously published study reports and has the potential to be less biased.^10^ ^11^ Additionally, such methods provide the opportunity to investigate questions which cannot be answered within individual trials or in systematic reviews, such as whether subgroups of children of different ages or in different countries respond to their vaccines differently.

In recent years it has become standard practice for clinical trial data to be made publically accessible through various online portals and pressure exists on all trialists to publish trial results promptly and in full. It is therefore feasible to conduct extensive individual participant data meta-analyses which were previously not possible, thus opening the door to new opportunities for research.
